# Incorporation of Pomegranate ( *Punica granatum* ) Extract Enhanced the Concentration of Bioactive Compounds and Shelf Life of Bread

**DOI:** 10.1002/fsn3.70690

**Published:** 2025-07-24

**Authors:** Nara Hellem Brazão Costa, Giovanna Biguetti Amadio, Y. J. Souza‐Santos, Rosemary Aparecida Carvalho, Fernanda Maria Vanin

**Affiliations:** ^1^ Laboratory of Bread and Dough Process (LAPROPAMA), Food Engineering Department, Faculty of Animal Science and Food Engineering (USP/FZEA) University of São Paulo Pirassununga São Paulo Brazil

**Keywords:** antioxidant activity, bioactive compounds, food and antifungal analysis, shelf life

## Abstract

Consumer demand for food without synthetic additives has considerably increased in recent years. The aim of this study was to evaluate the effect of pomegranate extract (PE) on bread shelf life and properties. PE was added at different concentrations: 10% (10PE), 12% (12PE), 15% (15PE), and 17% (17PE). Positive (PC) and negative (NC) control bread were also produced with calcium propionate and no antifungal additive, respectively. The breads were characterized in terms of physical properties, phenolic compounds content, antioxidant activity, and fungal and mold counts. No significant differences were observed among the samples in terms of water content, water activity (Aw), and pH during the 15‐day storage period. An increase in PE concentration resulted in a decrease in the specific volume and height of the breads. Bread containing PE exhibited significantly higher levels of phenolic compounds and antioxidant activity compared to the control formulations, and no fungal growth was observed throughout the 15‐day storage period.

## Graphical abstract

Pomegranate extract significantly increased bread's antioxidant capacity. Fungal growth was inhibited for up to 15 days of storage with pomegranate extract. Phenolic content was preserved even after baking and during storage.

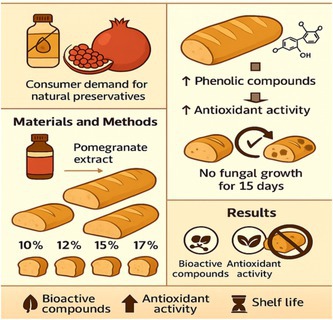

## 1 Introduction

Bread is rich in starch and complex carbohydrates, making it an important component of a balanced diet and a staple food consumed daily by billions of people worldwide (Rosell and Garzon 2015). Although bread has been consumed since the prehistoric period, one of the main challenges in bakery production remains the financial losses caused by microbial spoilage (Garcia et al. 2019).

Losses due to mold contamination are difficult to quantify, and there is currently no comprehensive estimate available. A study from 1993 reported that, in Europe, more than £200 million were lost annually due to fungal spoilage in bread (Legan 1993). In Brazil, data from 2011 indicated that approximately 11% of bread production was affected (Freire 2011).

In this context, synthetic preservatives play a crucial role in extending the shelf life of bread products (Axel et al. 2017). In recent years, there has been increasing pressure from both consumers and regulatory agencies to reduce the use of such additives in food products. However, under certain conditions, reducing the amount of preservatives used to prevent mold growth in baked goods can lead to a shorter shelf life (Magan and Aldred 2006).

Moreover, a major problem persisting in cereals and grain products is the presence of mycotoxins caused by fungal infections. The development of clean‐label alternatives is necessary due to the multiple drawbacks associated with traditional chemical preservatives (Axel et al. 2017). Numerous studies have demonstrated that by‐products from fruit and vegetable processing contain significant amounts of nutrients, minerals, vitamins, and phytochemicals, making them attractive candidates for use as food additives.

Therefore, the search for natural preservatives capable of extending the shelf life of bakery products has become a topic of growing interest. Pomegranate is widely recognized for its medicinal properties (Naz et al. 2007). Pomegranates have been shown to have a variety of biological properties, as well as antiviral, antibacterial, anticarcinogenic, and antioxidant properties (Gil et al. 2000; Malik et al. 2005; Reddy et al. 2007; Jurenka 2008; Duman et al. 2009). Different types of antioxidants, comprising anthocyanins, catechins, and ellagitannins, could be found on pomegranates, including in their peels, flowers, and juice (Kulkarni and Aradhya 2005; Ozgen et al. 2008; Duman et al. 2009; Hayrapetyan et al. 2012). These compounds play a key role in reducing spoilage by slowing the degradation processes in food products. Pomegranate cultivars have also demonstrated antimicrobial activities against various microorganisms (Braga et al. 2005; Reddy et al. 2007; Duman et al. 2009). Braga et al. (2005) showed that pomegranate extracts not only inhibited but also delayed the growth of *Staphylococcus aureus* and consequent enterotoxin production at concentrations of 0.01%, 0.05%, and 1% v/v. The fruit extracts also exhibited in vitro antibacterial activity against several tested bacterial strains (Meléndez and Capriles 2006).

Additionally, the incorporation of pomegranate extract can represent a strategy for aggregating compounds, increasing variety, and bioactivity, making them advantageous for use as food additives for nutritional enrichment (Czubaszek et al. 2023). Several bakery products already incorporate these extracts, including cookies and biscuits (Ismail et al. 2014, 2016; Srivastava et al. 2014; Maner et al. 2017; Kaderides et al. 2020), sponge cake (Mirab et al. 2020), and cake (Ayoubi et al. 2022), among others.

In addition, the use of different extracts in bread formulations has been explored in recent years. For example, Algboory et al. (2021) incorporated an aqueous extract of *Cyperus rotundus* rhizome to evaluate its effects on the quality and shelf life of wholemeal breads. The authors observed that the incorporation of the extract had no significant effect on bread water activity and water content, and the extract prolonged the shelf life of the bread by 7 days compared to the control formulation.

Pomegranate extract, in particular, has already found applications in various food products, such as meat products, nuggets as a functional additive (Bashir, Gilani, et al. 2016; Bashir, Rasool, et al. 2016), shrimp (Basiri et al. 2015), coconut oil to prevent oxidative processes (Bashir, Gilani, et al. 2016; Bashir, Rasool, et al. 2016), and to increase the antioxidant capacity of fermented milk (Chan et al. 2018).

Although limited, studies on the incorporation of extracts into bread are available in the literature. Given the reported antimicrobial and antioxidant properties of pomegranate residue extract, its use as an antimicrobial and nutritional enrichment agent in bread production emerges as a promising strategy to extend product shelf life. Therefore, the main objective of this study was to evaluate the effect of incorporating commercial pomegranate extract in the production of sliced bread, aiming to replace widely used chemical/synthetic additives/preservatives in the baking industry. Additionally, the study assessed the effects of the extract on the physical properties of the bread and the concentration of phenolic compounds during storage.

## 2 Material and Methods

### 2.1 Materials

Enriched wheat flour (Renata, Brazil), salt (Lebre, Brazil), refined sugar (Guanabara, Brazil), margarine (80% fat; Amélia, Brazil), dry biological yeast (Fleischmann, Brazil), and commercial pomegranate extract (Heide plant extracts, Brazil). Analytical grade chemical reagents were used to characterize the pomegranate extract and bread.

### 2.2 Methods

#### 2.2.1 Pomegranate Extract Characterization

##### 2.2.1.1 Total Phenolic Compound Content (TPC)

The total concentration of phenolic compounds in the extract was determined using the–Folin Ciocalteu method (Singleton et al. 1999). The samples were allowed to stand for 120 min in the absence of light, after which the absorbance was measured at 740 nm using a spectrophotometer (Thermo Scientific, Genesys 10S UV–Vis, USA). The results were converted into mg of gallic acid equivalents (mg GAE) per mL of extract.

##### 2.2.1.2 Evaluation of the Antioxidant Capacity of the Phenolic Extract

###### 2.2.1.2.1 FRAP—Ferric Reducing Antioxidant Power

The ferric reducing antioxidant power (FRAP) assay using the methodology described by Benzie and Strain (1996) was applied to evaluate the antioxidant potential of pomegranate extract. The absorbance of the samples was measured at a wavelength of 593 nm (Thermo Scientific, Genesys 10S UV–Vis, USA).

###### 2.2.1.2.2 ORAC—Oxygen Radical Absorbance Capacity

Using a spectrofluorometer (BMG Labtech, FLUOstar OPTIMA, Germany) the ORAC assay (Rodrigues et al. 2012) for antioxidant capacity was determined. The decay of fluorescein fluorescence in each well was monitored over 120 min, with excitation at 485 nm and emission at 528 nm.

###### 2.2.1.2.3 ABTS—(2,2′‐Azino‐Bis (3‐Ethylbenzothiazoline‐6‐Sulfonic Acid))

ABTS solution (7 mM) was prepared with 38.4 mg in distilled water in a volume of 10 mL in a volumetric flask. Separately, 378.4 mg of potassium persulfate was dissolved in 10 mL of distilled water to prepare the oxidizing solution. The ABTS+ radical was prepared by mixing 5 mL of the ABTS stock solution with 88 μL of potassium persulfate solution, the ABTS+ radical was then stored for 16 h and read at a wavelength of 734 nm on a spectrophotometer (Thermo Scientific, Genesys 10S UV–Vis, USA), and the result was calculated as a percentage of inhibition.

##### 2.2.1.3 Flavonoid Content

The total flavonoid content was determined using the method described by Zhishen et al. (1999), which involves reacting the samples with NaNO_2_, AlCl_3_, and NaOH. The absorbance was then measured at 510 nm using a spectrophotometer (Thermo Scientific, Genesys 10S UV–Vis, USA).

#### 2.2.2 Bread Making

Bread was produced according to the methodology proposed by Algboory et al. (2021), with some adaptations. The dough was prepared using 1 kg flour, 590 g water, 40 g sugar, 30 g fat (butter), 20 g salt, and 10 g dry biological yeast. As described by Łopusiewicz et al. (2023), pomegranate extract (PE) was added to the formulation, with its concentration calculated as a percentage of the total volume of water used in the bread formulation. Four bread formulations containing different concentrations of pomegranate extract were prepared: 10 g of the pomegranate extract/100 g of water (10PE), 12 g of the pomegranate extract/100 g of water (12PE), 15 g of the pomegranate extract/100 g of water (15PE), and 17 g of the pomegranate extract/100 g of water (17PE), in relation to the total of water used, as defined in preliminary tests. A control bread formulation was prepared without the addition of pomegranate extract (NC), and a second control formulation was prepared using a synthetic antimicrobial widely used in the market, calcium propionate (PC). All ingredients were mixed during 10 min using a high‐speed spiral mixer (AM‐12E; Famag Brasil, Brazil). The dough was then divided into 250 g portions and fermented in a fermentation chamber (Klimaquip‐CF‐20) at 37°C ± 2°C with 93% relative humidity for 120 min. The dough portions were placed into molds [20 cm (L) × 10 cm (W) × 6.5 cm (H)] and baked in an electric oven (Prática Techinipan) at 165°C for 25 min. After baking, the loaves were cooled at room temperature for 120 min, packed in 0.04 mm virgin polypropylene bags, and stored in a BOD (Marcondes‐MA415) at 25°C for 15 days. The breads were analyzed on days 1, 3, 6, 9, 12, and 15 after baking.

#### 2.2.3 Bread Characterization

##### 2.2.3.1 Aw and pH

Water activity (Aw) was determined using an AquaLab water activity meter (METER, AquaLab Series 3 TE) at 25°C. The pH was determined at 25°C ± 2°C using a pH meter (Kasvi, Benchotop phmeter W/ATC) according to the methodology described by AOAC (1995) (943.02).

##### 2.2.3.2 Water Content

Water content was determined according to AOAC (1995) (925.10). Samples were dried in an oven (Marconi, MA 033/1) at 105°C until a constant weight was obtained.

##### 2.2.3.3 Color

Color parameters (luminosity L*, a*, and b*) were evaluated using the CIELab scale with a colorimeter (Hunterlab, Miniscan EZ, USA) calibrated with white, black, and green standards (Bredariol et al. 2020).

##### 2.2.3.4 Specific Volume

Specific volume was determined according to the methodology described by Kim et al. (2013). The analysis was performed using a VolScan device (Stable Micro Systems, Godalming, UK) with a laser distance of 4 mm.

##### 2.2.3.5 Texture Profile

Texture profile analysis (TPA), including measurements of springiness, cohesiveness, and resilience, was determined according to the methodology described by Lara et al. (2011). Central slices of bread (25 mm thick) were analyzed using a texturometer (TA‐XT2i Stable Micro Systems Ltd., Surrey, UK) equipped with a 50 kg load cell.

##### 2.2.3.6 Bread Height

Bread height was measured before and after baking according to the methodology described by Wagner et al. (2008), using a digital caliper (MTX‐Matrix Tools for eXistence).

##### 2.2.3.7 Total Phenolic Compounds, Flavonoids, and Antioxidant Activity

Phenolic extract was performed following the methodology described by Dos Santos et al. (2015). Samples were shaken (300 rpm; 1 h; 25°C; Marconi, MA 420, Piracicaba, Brazil) and centrifuged (Eppendorf, 5430R, Germany) at 7830 rpm and 15°C for 10 min. The resulting extract was used to determine the total phenolic compounds, flavonoids, and antioxidant activity, as described for the characterization of Promegranta extract characterization (Section 2.2.1).

##### 2.2.3.8 Mold and Yeast Count

Mold and yeast counts were performed according to ISO 21527‐1:2008. Samples bread (9 g) were homogenized in 90 mL of buffered peptone water. From this, 1 mL aliquots were inoculated onto dehydrated culture medium plates (Compact Dry‐YMR, Nissui Pharmaceutical Co. Ltd.), incubated at 25°C for 48–72 h, and the number of colonies was counted.

#### 2.2.4 Statistical Analysis

Samples were analyzed by analysis of variance (ANOVA) with a 95% significance level (*p* < 0.05) and were statistically evaluated by the difference between the means using Duncan's test with a 95% confidence interval using SAS software (version 9.2, SAS Inc.).

## 3 Results and Discussion

### 3.1 Pomegranate Extract Characteristics

The pomegranate extract presented an average concentration of total phenolic compounds and flavonoids of 14.84 ± 52 mg of GAE/mL and 620.71 ± 18.82 mg of quercetin/mL, respectively (Table 1). Regarding antioxidant activity, the values obtained were 7.08 ± 0.85 umol TE/mL for the FRAP assay, 205.75 ± 8.58 umol TE/mL for the ABTS assay, and 38.85 ± 1.90 umol TE/mL for the ORAC assay (Table 1).

**TABLE 1:** Concentration of total phenolic compounds, flavonoids, and antioxidant activity of commercial pomegranate extract obtained by the FRAP, ABTS, and ORAC methods.

Analyze	Mean value
Total phenolics compounds (mg of ac gallic equivalent/mL)	14.84 ± 52
Flavonoids (mg of quercetin/mL)	620.71 ± 18.82
FRAP (umol te/mL)	7.08 ± 0.85
ABTS (umol te/mL)	205.75 ± 8.58
ORAC (umol te/mL)	38.85 ± 1.90

Cava and Ladero (2023) obtained an ethanolic pomegranate extract with similar characteristics, with a total phenolic compounds concentration of 19 mg GAE/mL. However, the flavonoid concentration reported by these authors (5 mg catechin eq/mL) was lower than that observed in the present study. These authors also evaluated the antioxidant potential of the pomegranate extract, reporting an ABTS activity of approximately 195 μmol TE.eq/mL, which was slightly lower than the value obtained in the present study. These differences may be attributed to the differences between the different parts of the pomegranate used to obtain phenolic extracts, in addition to the different methodologies used to extract the compounds.

### 3.2 Effect of Ethanolic Pomegranate Extract on the Physical Parameters of Bread

The effects of incorporating pomegranate extract (PE) and its concentration on the properties of sliced bread were evaluated. Furthermore, the effect of this extract on the shelf life of the product was evaluated. The following parameters were evaluated: pH, Aw, water content, crumb and crust color parameters, specific volume, final height, TPA, concentration of phenolic compounds, concentration of flavonoids, antioxidant activities, and mold and yeast counts.

**FIGURE 1:**
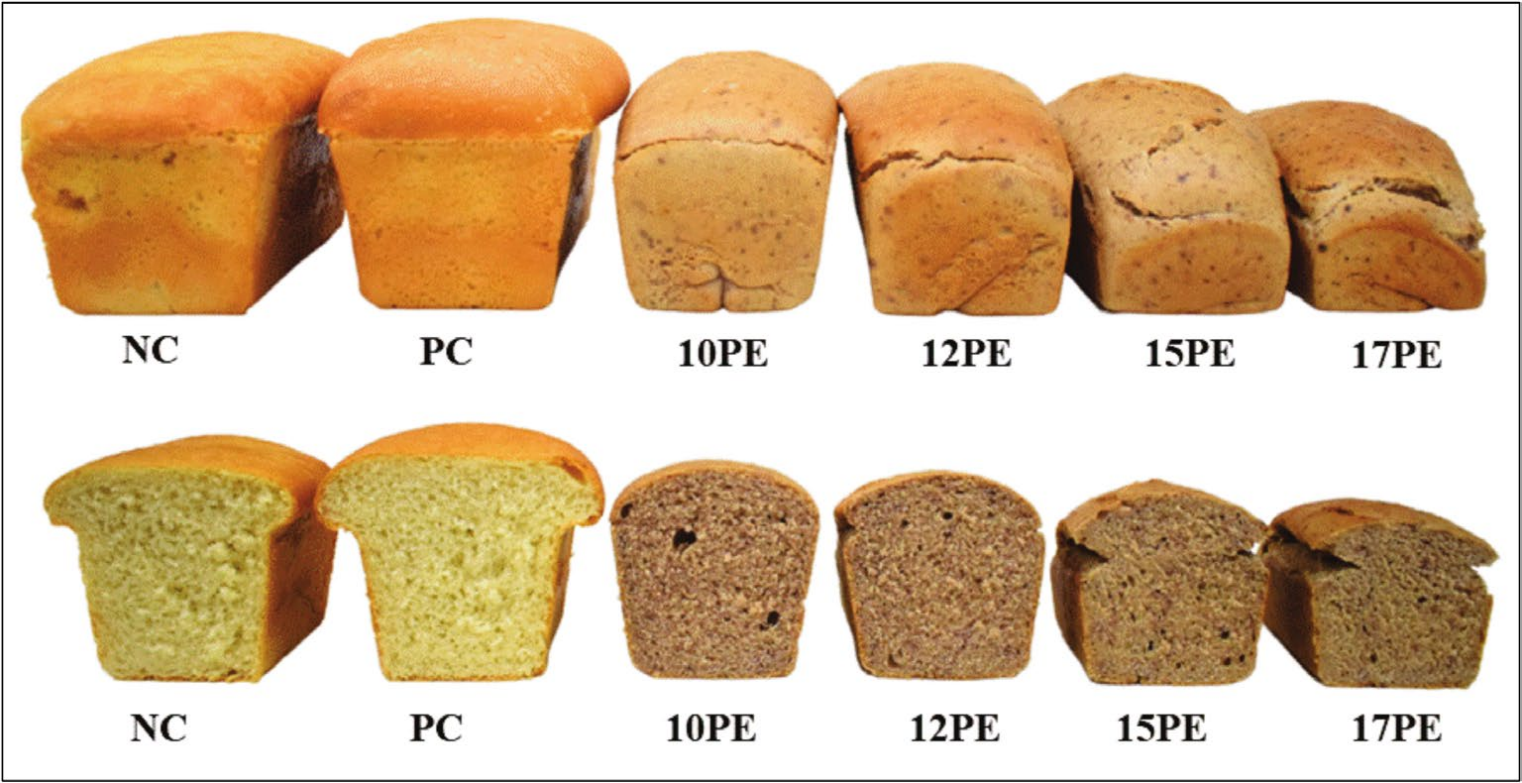
Images of different breads produced with different concentrations of pomegranate extract: Negative control (without pomegranate extract or other preservative) (CN), positive control (with calcium propionate) (CP), with 10 g of pomegranate extract/100 g of water (10PE), with 12 g of pomegranate extract/100 g of water (12PE), with 15 g of pomegranate extract/100 g of water (15PE) and with 10 g of pomegranate extract/100 g of water (17PE).

For the pH, Aw, crumb and crust color, specific volume, and final height, no significant variations were observed throughout the storage period. Therefore, only the results obtained on the first day are presented (Table 2). Examples of breads produced under different formulation conditions are shown in Figure 1.

**TABLE 2:** Effect of pomegranate extract concentration on the physical parameters on bread properties: Negative control bread—NC (without antimicrobial additive), positive control bread—PC with calcium propionate, with 10 g of pomegranate extract/100 g of water (10PE), with 12 g of pomegranate extract/100 g of water (12PE), with 15 g of pomegranate extract/100 g of water (15PE) or with 10 g of pomegranate extract/100 g of water (17PE).

Property	Bread formulation					
	NC	PC	10PE	12PE	15PE	17PE
pH	5.41 ± 0.01b	5.44 ± 0.01b	5.81 ± 0.11a	5.66 ± 0.07a	5.64 ± 0.13a	5.65 ± 0.30a
Aw	0.94 ± 0.01a	0.94 ± 0.01a	0.93 ± 0.01b	0.94 ± 0.01a	0.94 ± 0.01a	0.94 ± 0.01a
Water content (g water/100 g of bread)	50.7 ± 2.2ab	53.2 ± 2.9a	51.1 ± 3.9ab	49.0 ± 1.1b	49.8 ± 1.8b	49.7 ± 2.9b
Specific volume (mL/g)	2.52 ± 0.32b	3.10 ± 0.29a	1.77 ± 0.20c	1.80 ± 0.03c	1.52 ± 0.06d	1.41 ± 0.07d
Height (mm)	11.2 ± 3.4a	12.7 ± 3.7a	11.5 ± 4.9a	10.3 ± 4.7a	6.6 ± 3.9b	−1.1 ± 2.9c
*Crumb color*						
L*	76.22 ± 2.65a	75.35 ± 2.92a	60.65 ± 3.20c	64.04 ± 1.52b	61.39 ± 1.54bc	62.67 ± 2.11bc
a*	1.62 ± 0.48d	1.43 ± 0.48d	5.14 ± 0.86a	3.95 ± 0.21bc	4.06 ± 0.22b	3.45 ± 0.33c
b*	21.40 ± 0.64c	21.56 ± 0.67c	20.70 ± 2.30c	23.27 ± 0.39b	25.58 ± 0.62a	25.65 ± 0.89a

Means followed by different lowercase letters on the same line indicate statistical difference between formulations.

Table 2 summarizes the physical characteristics of bread.

In general, Aw, pH, and water content values of the breads were not significantly affected by the addition of pomegranate extract.

Similar findings were reported by Algboory et al. (2021), who also observed no significant effect on the water activity of bread produced with different concentrations of *C. rotundus* aqueous extract. However, these authors observed a significant reduction in pH values with the addition of the aqueous extract of *C. rotundus* in the formulation. Likewise, the use of hop extract did not cause significant variations in the pH and Aw values of bread (Nionelli et al. 2018).

Regarding water content results, other studies involving different extracts, such as the aqueous extract of *C. rotundus* (Algboory et al. 2021), green coffee extract (Mukkundur Vasudevaiah et al. 2017), and black tea extract fractions (Culetu et al. 2018) also reported no significant effects on the water content of bread.

The incorporation of pomegranate extract significantly affected crumb color, decreasing the lightness (L*), and increasing both a* and b* values (Table 2). These color changes are also visually apparent in the images presented in Figure 1. Similar trends have been observed in breads formulated with green coffee extract, where increasing extract concentration significantly altered L*, a*, and b* values (Mukkundur Vasudevaiah et al. 2017). Likewise, the crumb of bread produced with fractions of black tea extract at different concentrations (crumb) (Culetu et al. 2018) and the crust of bread made with hop extract (crust) (Nionelli et al. 2018) showed significant variation in color parameters.

**FIGURE 2:**
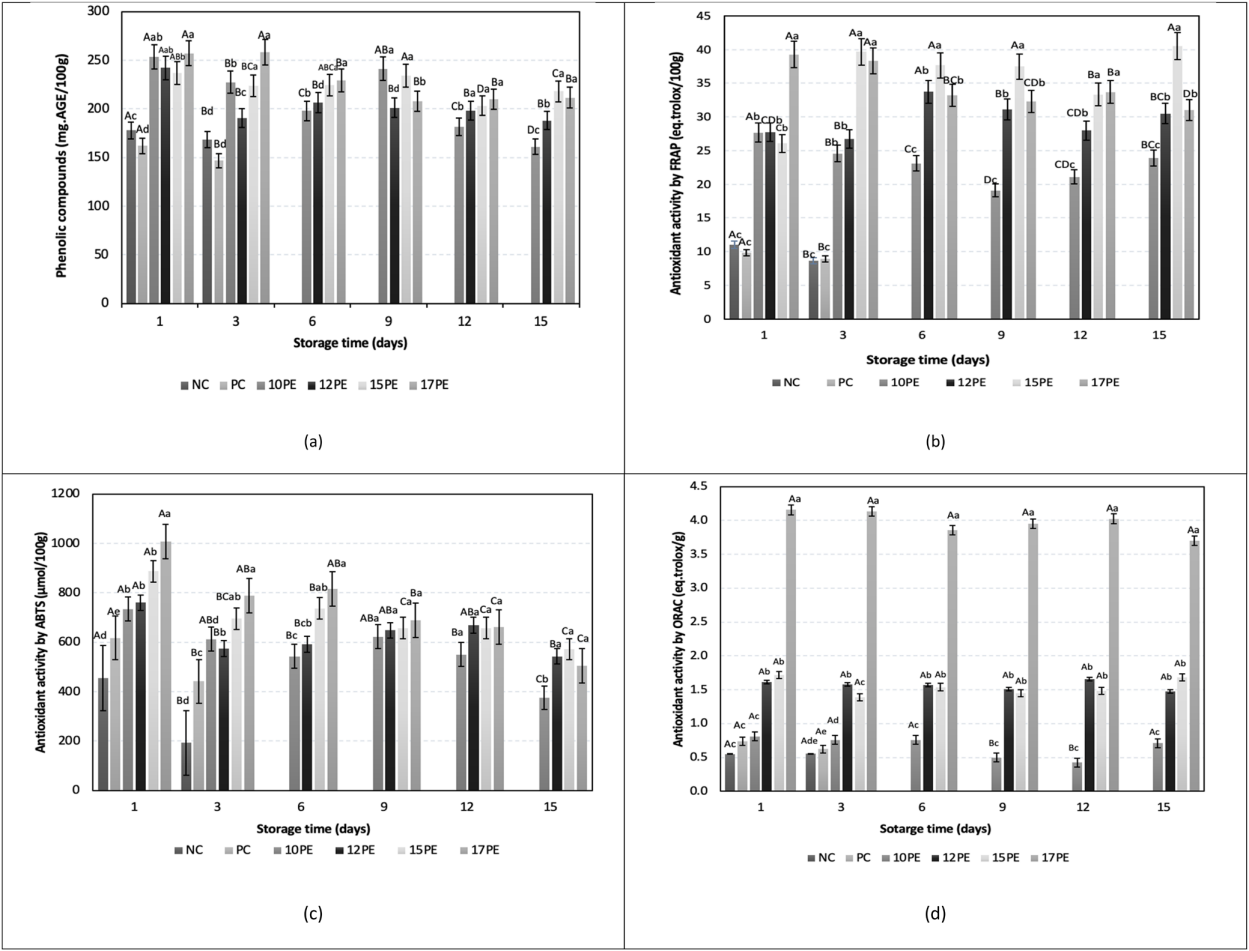
Phenolic compounds concentration (a), antioxidant activity by FRAP (b), ABTS (c), and ORAC (d) for breads produced with different concentrations of pomegranate extract: Negative control bread—NC (without antimicrobial additive), positive control bread—PC with calcium propionate with 10 g of pomegranate extract/100 g of water (10PE), with 12 g of pomegranate extract/100 g of water (12PE), with 15 g of pomegranate extract/100 g of water (15PE) and with 10 g of pomegranate extract/100 g of water (17PE). Means followed by different capital letters indicate a statistical difference between the means over the storage period for the same formulation. Means followed by different lowercase letters indicate statistical differences between formulations for the same storage time.

Both the specific volume and final height of the breads were significantly affected by increasing pomegranate extract concentration (Table 2). Breads containing calcium propionate (PC) (synthetic antifungal) exhibited the highest specific volume and final height, differing significantly from all formulations containing pomegranate extract and the negative control (NC), formulation without added extract (Table 2, Figure 2). Furthermore, a progressive and significant decrease (*p* < 0.05) in specific volume and final height was observed with increasing PE concentration.

Similar reductions in specific volume have been reported for breads produced with different concentrations of black tea extract (Culetu et al. 2018). In contrast, increasing green coffee extract concentrations resulted in a significant increase in bread volume (Mukkundur Vasudevaiah et al. 2017). According to these authors, the volume increase could be attributed to the extract's positive effects on dough tenacity and extensibility balance, as indicated by alveographic data (P/L ratio), and to enhanced starch swelling during baking.

Bread weight, closely linked to the baking process and final volume, showed that the highest height values were recorded for the control formulations (NC and PC), as well as for 10PE and 12PE (Table 2). The 17PE formulation showed a significantly negative reduction compared to the other PE‐containing formulations. This reduction can be attributed to the higher PE concentration, which may have impaired fermentation and oven spring, resulting in a final height (post‐baking) even lower than the pre‐fermentation height. Mata‐Ramírez et al. (2018) observed a similar effect when they evaluated the effect of incorporating *hibiscus sabdariffa* into wheat bread, that is, a decrease in the height of the bread due to an increase in incorporation.

From the results reported in the current study, it can be concluded that incorporating pomegranate extract within the tested concentration range studied did not cause significant changes in the pH, Aw, and water content values of the breads, which may represent a positive aspect. However, the specific volume and final height values of the products showed a significant reduction, which may in turn have impacted some texture parameters (see Section 3.3.1).

### 3.3 Effect of Pomegranate Extract on Bread Shelf Life

#### 3.3.1 Texture Profile Analyses

The texture profile parameters, elasticity, cohesiveness, and resilience of the breads are presented in Table 3.

**TABLE 3:** Texture profile analysis parameters, elasticity, cohesiveness, and resilience, of breads produced with different pomegranate extract concentrations: Negative control bread—NC (without antimicrobial additive), positive control bread—PC (with calcium propionate), with 10 g of pomegranate extract/100 g of water (10ER), with 12 g of pomegranate extract/100 g of water (12ER), with 15 g of pomegranate extract/100 g of water (15ER) and with 10 g of pomegranate extract/100 g of water (17ER).

Property	Storage time (days)					
	1	3	6	9	12	15
*Elasticity*						
NC	0.778 ± 0.11Aa	0.772 ± 0.04Aabc	—	—	—	—
PC	0.829 ± 0.09Aa	0.735 ± 0.07Ac	—	—	—	—
10PE	0.827 ± 0.02Aa	0.754 ± 0.05Babc	0.758 ± 0.08Ba	0.712 ± 0.05Bab	0.749 ± 0.05Ba	0.742 ± 0.06Ba
12PE	0.831 ± 0.02Aa	0.822 ± 0.02Aa	0.822 ± 0.03Aa	0.761 ± 0.05Ba	0.739 ± 0.04Ba	0.730 ± 0.05Ba
15PE	0.794 ± 0.03Aa	0.792 ± 0.11Aab	0.742 ± 0.06ABCa	0.665 ± 0.08Cb	0.700 ± 0.04Aa	0.773 ± 0.07ABa
17PE	0.769 ± 0.03Aa	0.707 ± 0.03Abc	0.657 ± 0.08Bb	0.741 ± 0.06Aa	0.729 ± 0.09ABa	0.701 ± 0.04ABa
*Cohesiveness*						
NC	0.672 ± 0.02Aa	0.500 ± 0.09Ba	—	—	—	—
PC	0.553 ± 0.10Ac	0.440 ± 0.04Bab	—	—	—	—
10PE	0.677 ± 0.04Aa	0.425 ± 0.03Bb	0.381 ± 0.02BCab	0.370 ± 0.03Ca	0.366 ± 0.02Cab	0.362 ± 0.07Cab
12PE	0.606 ± 0.04Abc	0.399 ± 0.03Cb	0.450 ± 0.08Ba	0.388 ± 0.02Ca	0.380 ± 0.03Cab	0.369 ± 0.03Ca
15PE	0.619 ± 0.03Aab	0.440 ± 0.07Bab	0.421 ± 0.07Bab	0.410 ± 0.13Ba	0.439 ± 0.15Ba	0.361 ± 0.05Bab
17PE	0.584 ± 0.02Abc	0.407 ± 0.03Bb	0.354 ± 0.05Cb	0.350 ± 0.04Ca	0.333 ± 0.04Cb	0.312 ± 0.02Cb
*Resilience*						
NC	0.302 ± 0.03Aa	0.189 ± 0.04Ba	—	—	—	—
PC	0.237 ± 0.08Aa	0.164 ± 0.02Aab	—	—	—	—
10PE	0.296 ± 0.04Aa	0.139 ± 0.01Bb	0.126 ± 0.01Bb	0.127 ± 0.01Ba	0.125 ± 0.01Ba	0.123 ± 0.03Ba
12PE	0.255 ± 0.02Aab	0.141 ± 0.02BCb	0.164 ± 0.04Ba	0.137 ± 0.01BCa	0.137 ± 0.02Ca	0.138 ± 0.01BCa
15PE	0.258 ± 0.03Aab	0.163 ± 0.04Bab	0.162 ± 0.03Bab	0.157 ± 0.05Ba	0.167 ± 0.06Ba	0.143 ± 0.02Ba
17PE	0.227 ± 0.02Ab	0.146 ± 0.01Bb	0.126 ± 0.03BCb	0.129 ± 0.01BCa	0.130 ± 0.02BCa	0.120 ± 0.01Ca

*Note:* Means followed by different capital letters on the same line indicate a statistical difference between the means over the storage period for the same formulation. Means followed by different lowercase letters in the same column indicate statistical differences between formulations for the same storage time.

Elasticity reflects the capacity of the deformed material to return to its original shape after the removal of the deforming force. For all bread samples analyzed, it was observed that the use of pomegranate extract and increasing its concentration in the bread formulation did not have a significant effect on the elasticity values of the breads (Table 3). However, regarding the effect of storage time, a significant reduction in the elasticity values of breads produced with 10% and 12% pomegranate extract was observed from the sixth day of storage. In contrast, breads with higher concentrations (15% and 17%) showed no significant reduction in the elasticity values during the storage period.

The addition of pomegranate extract had a significant impact on bread cohesiveness on the first day of analysis (Table 3). Despite these statistical differences, the cohesiveness values remained within the range observed for both the negative control (NC) and positive control (PC) breads. Furthermore, significant reduction in cohesiveness was observed in all formulations, including both controls and PE‐containing breads, starting from the third day of storage.

Similar results were reported by Culetu et al. (2018) who observed reductions in both elasticity and cohesiveness during the storage of bread enriched with black tea extract, resulting in a less elastic and cohesive crumb.

Regarding resilience, no significant differences were observed among the PC, NC, and 10PE breads after 1 day of storage (Table 3). In contrast, breads with PE concentrations above 10% presented significantly lower resilience values compared to the control samples (Table 3). Despite these initial differences, resilience values across all formulations did not differ significantly after the third day of storage. These findings are consistent with those reported by Torgbo et al. (2022) for breads formulated with rambutan peel extract.

The interaction between the PE formulation's components and the dough matrix, gluten, starch, sugar, and fat content may have also influenced the firmness (Sun‐Waterhouse et al. 2011). The molecular redistribution of water between mainly starch and gluten and added extracts can affect water mobility and compromise the development and stability of the gluten network.

#### 3.3.2 Total Phenolic Compounds and Flavonoids Concentration

In general, the incorporation of pomegranate extract into the bread formulation resulted in a significant increase in the concentration of total phenolic compounds in the products (Figure 2a). As expected, higher concentrations of pomegranate extract led to a proportional and significant increase in the total phenolic concentration (Figure 2a). Significant differences among the bread formulations were observed throughout the storage period with respect to total phenolic compound levels. Notably, the 17PE formulation consistently exhibited significantly higher total phenolic content compared to the other formulations during the storage. The concentration of phenolic compounds in breads with PE, 10PE, 12PE, 15PE, and 17PE can be considered satisfactory because these compounds have thermal and oxidative sensitivity and could therefore suffer from the effects of the baking and storage processes. Despite this, the phenolic activity of the breads enriched with the extract remained high (Figure 2a).

Flavonoid concentrations could not be quantified in the NC, PC, 10PE, 12PE, and 15PE. Only the formulation produced with 17PE presented an initial flavonoid concentration of 2180.1 mg quercetin/100 g dry sample (Table 4). However, from the third day of storage, a significant reduction in flavonoid content was observed in breads formulated with 17PE.

**TABLE 4:** Flavonoids and colony forming unit values (CFU) verified for breads produced with different concentrations of pomegranate extract: Negative control bread—NC (without antimicrobial additive), positive control bread—PC (with calcium propionate), with 10 g of pomegranate extract/100 g of water (10PE), with 12 g of pomegranate extract/100 g of water (12PE), with 15 g of pomegranate extract/100 g of water (15PE) and with 10 g of pomegranate extract/100 g of water (17PE).

Bread formulation	Storage time (days)			
	1	3	6	15
*Flavonoids* (*mg quercetin/100 g dry sample*)				
NC	ND	ND	—	—
PC	ND	ND	—	—
10PE	ND	ND	ND	ND
12PE	ND	ND	ND	ND
15PE	ND	ND	ND	ND
17PE	2180.1a	1769.5b	1538.5b	1027.0c
*CFU*				
NC	ND	ND	7.5 × 10^5^ ± 3.79a	—
PC	ND	ND	3.1 × 10^6^ ± 1.00b	—
10PE	ND	ND	ND	ND
12PE	ND	ND	ND	ND
15PE	ND	ND	ND	ND
17PE	ND	ND	ND	ND

*Note:* Means followed by different lowercase letters indicate statistical differences between formulations.

Abbreviation: ND, not detected.

It is important to highlight that several studies have explored the inclusion of phytonutrients in bread formulations to enhance their nutritional value. Breads supplemented with Roselle ( *Hibiscus sabdariffa* ) (Mata‐Ramírez et al. 2018), Rambutan (*Nephelium lappaceum L*.) peel extract (Torgbo et al. 2022), and *Cyperus rotundus* rhizome aqueous extract (Algboory et al. 2021) also showed increased total phenolic content with the addition of higher extract concentrations. The total polyphenol content of breads produced with green coffee extract was significantly higher than that of control bread (Mukkundur Vasudevaiah et al. 2017). Likewise, the use of hop extract (Nionelli et al. 2018) and black tea extract fractions (Culetu et al. 2018) also resulted in breads with total phenolic content improved compared to control breads.

#### 3.3.3 Antioxidant Activity

The antioxidant activity of the bread formulations supplemented with PE was evaluated using FRAP, ABTS, and ORAC assays (Figure 2). The addition of PE increased the antioxidant activity of the bread. For all methods evaluated, the higher the PE concentration, the higher the antioxidant activity of the breads (Figure 2). The increase in antioxidant activity values in breads with PE (10PE, 12PE, 15PE, and 17PE) compared to the control formulations (NC and PC) can be attributed to the incorporation of phenolic compounds and, particularly for the flavonoids in the 17PE formulation. These results are in agreement with those reported by Torgbo et al. (2022) for breads produced using rambutan peel extract.

Interestingly, the antioxidant capacity of breads with 17PE was approximately 8–9 times higher than that of control bread when FRAP and ORAC were used (Figure 2b,d).

These results indicate that a significant portion of the bioactive compounds from the pomegranate extract retained their functional properties even after exposure to baking temperatures. This highlights the potential of pomegranate extract as a functional ingredient for developing breads and other bakery products with enhanced health benefits, providing valuable information for consumers, nutritionists, and food manufacturers.

#### 3.3.4 Mold and Yeast Counts

No mold or yeast growth was observed in any of the bread formulations up to the third day of storage (Figure 1). Visual mold growth was observed on the surfaces of the NC and PC breads by the sixth day (Figure 1). This effect was confirmed by microbiological analyses (Table 4), which showed that the fungal counts in both the NC and PC groups exceeded the detection limit on day 6 of storage. In contrast, breads enriched with PE, regardless of concentration, showed no detectable fungal growth during the same period.

The addition of PE inhibited the growth of fungi in bread and extended the shelf life until the 15th day of storage. Bao et al. (2023) added star anise extract to sliced bread with the aim of inhibiting the growth of fungi of the genus *Penicillium roqueforti* and *Aspergillus niger* and found that the addition of the extract prolonged the shelf life by 6 days.

In another study, Culetu et al. (2018) observed daily visible mold growth in control breads but noted no mold development in breads enriched with black tea extracts until the eighth day of storage. Although no significant differences (*p* > 0.05) in yeast and mold counts were found among formulations within the first 3 days, visible mold appeared on the surface of control breads by day 4.

According to Sharayei et al. (2020) the antifungal activity of pomegranate peel extract is primarily attributed to its phenolic compounds, which possess well‐documented antimicrobial properties.

In this context, the results observed in the current study demonstrated that PE was effective in inhibiting the growth of spoilage microorganisms in bread, compared to that reported in the literature, with a survival of at least 7 days compared to previously published studies.

## 4 Conclusions

Based on the results presented, it can be concluded that the pomegranate extract (PE) effectively inhibited fungal growth in the breads, with no fungal growth over the 15‐day storage period.

The incorporation of PE into the bread formulation significantly increased the bioactive compounds present in the bread and enhanced the antioxidant capacity compared to the NC and PC formulations.

However, the presence of PE in bread formulations also led to changes in certain physical properties, such as texture and color, which may potentially impact consumer sensory acceptance.

Overall, the use of pomegranate extract presents a promising strategy for extending the shelf life of breads, while improving its bioactive profile, offering a natural and safe alternative to synthetic additives. These findings provide valuable insights for the food and bakery industries seeking to develop cleaner‐label products with health‐promoting properties.

Future studies are recommended to explore the use of lower concentrations of PE to minimize undesirable changes in bread physical properties, as well as to assess consumer acceptability and sensory perception of the final product.

## Author Contributions

**Nara Hellem Brazão Costa:** conceptualization (lead), data curation (lead), formal analysis (lead), methodology (lead), writing – original draft (lead), writing – review and editing (lead). **Giovanna Biguetti Amadio:** conceptualization (equal), data curation (equal), formal analysis (equal), methodology (equal), writing – original draft (equal). **Y. J. Souza‐Santos:** investigation (equal), methodology (equal), visualization (equal), writing – original draft (equal), writing – review and editing (equal). **Rosemary Aparecida Carvalho:** formal analysis (equal), investigation (equal), methodology (equal), validation (equal), writing – original draft (equal), writing – review and editing (equal). **Fernanda Maria Vanin:** conceptualization (equal), data curation (equal), funding acquisition (equal), investigation (equal), methodology (equal), project administration (equal), resources (equal), supervision (equal), validation (equal), visualization (equal), writing – original draft (equal), writing – review and editing (equal).

## Conflicts of Interest

The authors declare no conflicts of interest.

## Data Availability

The data that support the findings of this study are available on request from the corresponding author.
